# Genetic Variation and Association with Post-Operative Outcomes for Neonates and Infants in the Cardiac Intensive Care Unit

**DOI:** 10.3390/genes17080910

**Published:** 2026-07-31

**Authors:** Danielle Devine, Aaron Tien, Haoting He, Tracy Baust, Rod Ghassemzadeh, Jiuann-Huey Ivy Lin

**Affiliations:** 1 Department of Critical Care Medicine, School of Medicine, University of Pittsburgh, Alan Magee Scaife Hall, Suite 600, 3550 Terrace Street, Pittsburgh, PA 15213, USA; tbasust@childrensnational.org (T.B.); rod.ghassemzadeh@upmc.edu (R.G.); 2 Division of Pediatric Cardiac Critical Care, UPMC Children’s Hospital of Pittsburgh, Suite 4552, 4401 Penn Ave, Pittsburgh, PA 15224, USA; 3 Department of Statistics, Kenneth P. Dietrich School of Arts and Sciences, University of Pittsburgh, 1826 Wesley W. Posvar Hall, 230S Bouquet Street, Pittsburgh, PA 15260, USA; aat64@pitt.edu; 4 Department of Cell Biology, School of Medicine, University of Pittsburgh, 3500 Terrace Street, Pittsburgh, PA 15261, USA; hah94@pitt.edu

**Keywords:** copy number variants, microarray, outcomes, pediatric cardiac critical care

## Abstract

Introduction: Congenital heart defects (CHD) occur in 1% of live births, with an estimated at least 33% of affected infants having genetic defects. It has become standard to screen children with CHD for genetic findings that could aid clinical decision-making, yet modern testing yields large volumes of information without understanding clinical utility. Genome-wide studies have identified associations between large copy number variants and neurocognitive outcomes in patients with CHD. Still, little research exists on the prognostic power of screening tests such as microarrays. The goal of this study was to determine if abnormal microarray results in infants with CHD were associated with worse clinical outcomes in the cardiac intensive care unit (CICU). Method: This was a single-center retrospective cohort study. The Society of Thoracic Surgery (STS) and Pediatric Cardiac Critical Care (PCICU) registries (PC4) were queried for all surgical admissions of neonates and infants between 1 January 2014 and 31 December 2019. Patient demographics, surgical details, and post-operative outcomes were collected for surgical admissions related to the patient’s index operation. A chart review was performed to ascertain microarray results, further genetic testing, and longitudinal outcomes. Patients without microarray results or long-term institutional follow-up were excluded. Wilcoxon rank-sum and chi-square tests were used to compare outcomes defined and collected in the PC4 registry between patients with normal and abnormal microarray results. Subgroup analysis was then performed using the same outcome measures to compare normal and abnormal microarray groups in children who required cardiac surgeries within 30 days of life and in children who presented with ventricular septal defect (VSD), atrioventricular septal defect (AVSD), aortic stenosis (AS), or Tetralogy of Fallot (TOF). Results: Of the 412 infants with surgical admissions to the CICU between 2014 and 2019, 43 infants had no longitudinal follow-up, and 65 infants had no microarray results. Three hundred and four infants were included in this study, of which 196 children had normal microarrays and 108 infants had abnormal microarrays. Both groups had similar distributions of gestational age and birth weight, but fundamental diagnoses and primary procedures differed significantly between groups (*p* < 0.005). STS score, deep hypothermic circulatory arrest (DHCA) time, and bypass time were each significantly lower in children with abnormal microarrays than in those with normal microarrays across the study. The patients with abnormal microarrays had a higher incidence of gastrostomy (G)-tube placement during their lifetime than infants with normal microarrays. On subgroup analysis, despite the differences in fundamental diagnoses and the fact that primary procedures differed significantly between normal and abnormal microarray groups that required cardiac surgery within 30 days of life, there were no detectable differences in the surgical and PCICU outcomes. DHCA time was once again significantly lower in the abnormal microarray subgroup in the second subgroup analysis. Conclusions: Our data indicate that microarray results have limited value in predicting immediate post-operative outcomes, but children with copy number variants should be included in future CHD research.

## 1. Introduction

Congenital heart disease (CHD) occurs in up to 1% of live births [1,2], with an estimated 33% to 46% of affected infants having genetic disorders [3,4]. It has become standard to screen children with CHD for genetic findings that could aid in clinical decision-making, yet modern testing yields large volumes of information without an understanding of clinical utility [4,5,6,7,8,9]. Genome studies in certain subsets of CHD patients have identified associations between large copy number variants (CNVs) and long-term outcomes such as impaired transplant-free survival and inferior neurocognitive outcomes [10,11]. Still, little data exists on how large CNVs affect immediate care and short-term post-operative outcomes [11,12,13,14]. Furthermore, many of these studies use research-grade sequencing and genotyping tools that are not yet universally available. In the clinical setting, although rapid whole-exome sequencing is now a first-line tool, microarray studies have long been used as a first-line genetic screening tool for individuals with CHD [15,16]. While multiple studies have investigated the diagnostic value of microarray findings in relation to phenotypic manifestations of CHD, only a few studies have examined clinical outcomes of children with cardiac disease in the setting of positive microarray results [17,18]. With the continued advances in genetic testing and the expansion of genetic information available at our fingertips, research is needed to explore the prognostic information encoded in these findings. The goal of this study was to determine if abnormal microarray results in neonates and infants with CHD were associated with worse post-operative clinical outcomes in the pediatric cardiac intensive care unit (PCICU).

## 2. Methods

This was a single-center retrospective cohort study. The Society of Thoracic Surgery (STS) and Pediatric Cardiac Critical Care (PC4) databases were queried for all admission encounters of neonates and infants less than or equal to 1 year of age who underwent cardiac surgery requiring cardiopulmonary bypass (CBP) between 1 January 2014 and 31 December 2019. Patient demographics, surgical details, and post-operative outcomes were collected for surgical admissions related to a patient’s index operation. A chart review was performed to ascertain microarray results, further genetic testing, and longitudinal outcomes. Chromosomal microarray analysis was performed either by array comparative genomic hybridization or using a single nucleotide polymorphism (SNP) array [19]. The whole-genome chromosomal microarray result was a dichotomous variable classified as either normal or abnormal. The abnormal chromosomal microarray results occurred when one or more missing or extra chromosomal segments were present [20] or when the region of homozygosity (ROH) was >1 Mb [16]. Patients without microarray results or long-term institutional follow-up were excluded. Wilcoxon rank-sum and chi-square tests were used to compare outcomes between patients with normal and abnormal microarray results. A secondary analysis correcting for the STAT Mortality category (Society of Thoracic Surgeons: European Association for Cardio-Thoracic Surgery Congenital Heart Surgery Mortality Category) was then performed, with STAT categories 4 and 5 combined into a single group. Additionally, two subgroup analyses were performed. The first investigated the difference in outcomes between children with abnormal and normal microarrays who required their index surgery in the first 30 days of life, the definition of critical congenital heart defects [21]. The second analysis was performed on infants and neonates who presented with ventricular septal defect (VSD), atrioventricular septal defect (AVSD), aortic stenosis (AS), or Tetralogy of Fallot (TOF), the group with the most common primary diagnoses among infants with abnormal microarrays.

## 3. Statistics

All statistical analyses were conducted using R version 4.1.0. Analyses for categorical explanatory variables, such as mortality at discharge, were conducted using Pearson chi-squared tests using the R chisq.test() function. For the variables that violated the assumptions of the test, a Pearson chi-square test with Yates’ continuity correction was conducted using the same R function. Continuous explanatory variables, such as PCICU length of stay (LOS), were analyzed using Wilcoxon rank-sum tests using the R wilcox.test() function. We used a non-parametric test, either because the distribution of the variable in the sample was skewed or because we did not feel comfortable making the assumptions necessary for a parametric test such as a *t*-test. The summary () function was used to check for not applicable (NA) values and provide a rudimentary numerical summary for the data. NA values were handled by setting them to 0. We used a lower *p*-value to avoid the multiple comparisons fallacy (*p* ≤ 0.01). To compare differences between the normal and abnormal microarray groups for each outcome of interest while adjusting for STAT category, we fitted logistic regression models for categorical outcomes and linear regression models for continuous outcomes. In each model, the outcome of interest was the response variable, and STAT category and microarray group were included as predictor variables.

## 4. Results

**Figure 1:**
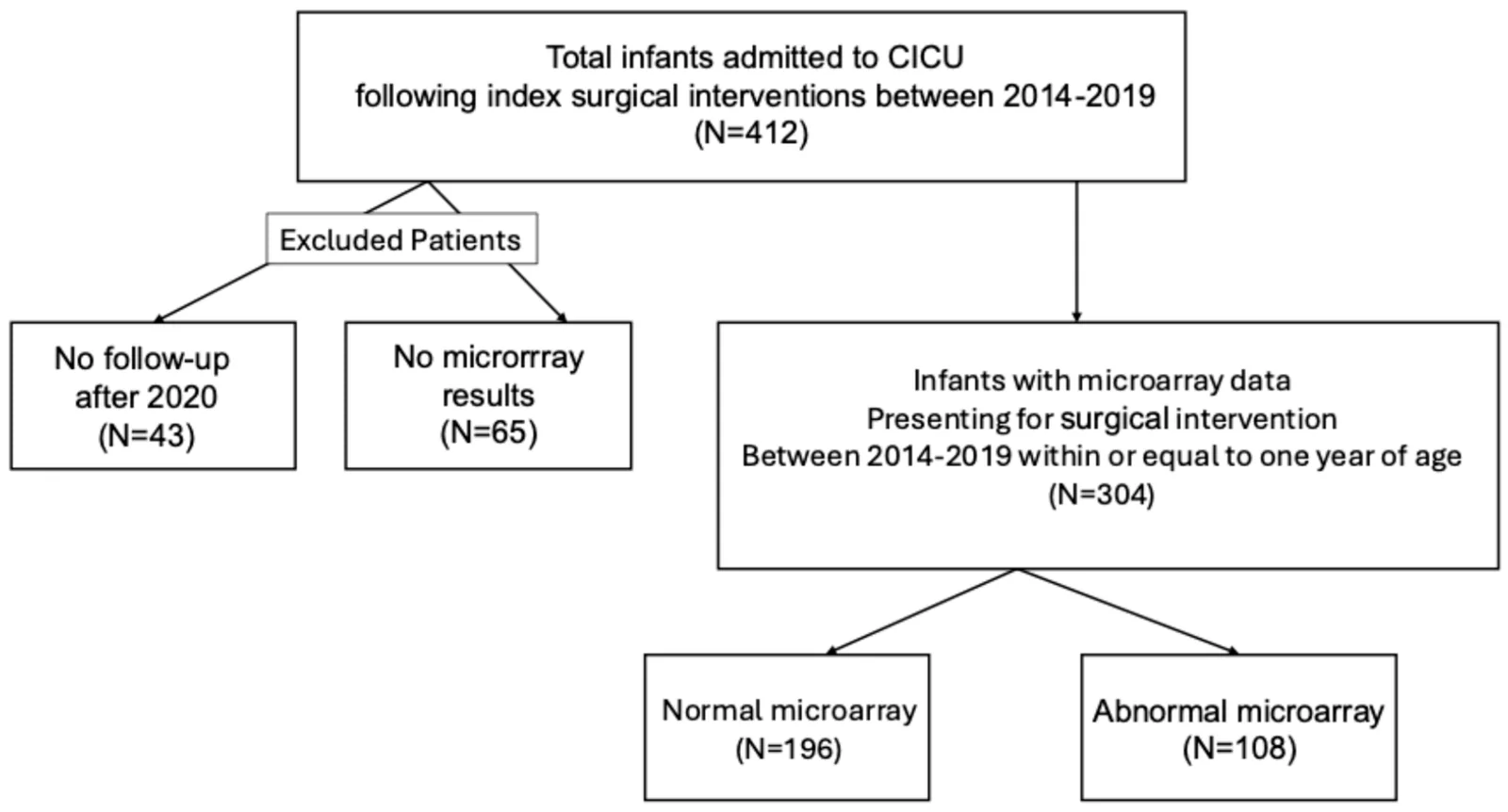
Inclusion criteria.

Of the 412 neonates and infants who underwent their initial cardiac surgery requiring CPB and subsequent admission to the CICU between 2014 and 2019, 43 had no longitudinal follow-up, and 65 had no microarray results. Of note, microarray data were frequently missing for children diagnosed postnatally by their primary care provider. The remaining 304 patients were included in this study [Figure 1]. Table 1 presents demographic information for the 304 patients included in this study, of which 196 neonates and infants had normal microarray results, and 108 (36%) had abnormal results [Supplemental Tables S1 and S2]. Forty-one percent of included neonates and infants were female, and 83% were born between 37 and 40+ weeks of gestational age. Further, 36% of patients with abnormal microarrays had extracardiac anomalies, compared with 22% of patients with normal microarrays (*p* = 0.03). The most common primary diagnoses among infants with abnormal microarrays were VSD (N = 20), TOF (N = 14), AVSD (N = 26), and aortic hypoplasia/stenosis (N = 10). The four most commonly occurring primary diagnoses in infants with normal microarrays include transposition of the great arteries (N = 21), TOF (N = 21), aortic hypoplasia/stenosis (N = 30), and single right-dominant ventricle (HLHS) (N = 34). There is a significant difference between the primary diagnosis and primary procedures between the normal and abnormal microarray groups. The individuals in the abnormal microarray group had less complex cardiac anatomy and underwent fewer complex procedures (*p* < 0.005).

**Figure 2:**
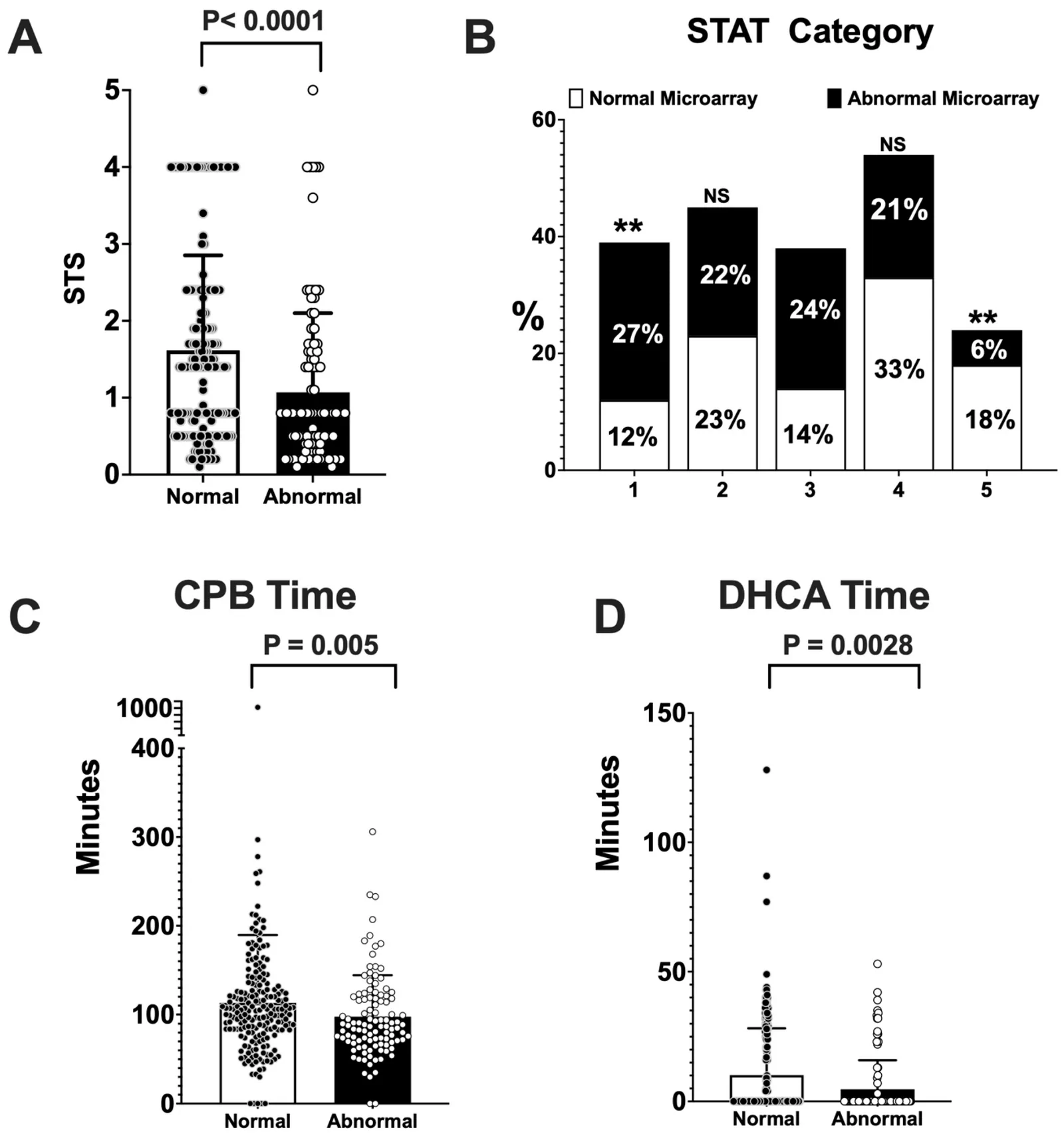
Box-and-whisker plots representing statistically significant outcomes between normal and abnormal microarray groups in the whole patient cohort. (**A**) STS score, (**B**) STAT Category, (**C**) Cardiopulmonary bypass (CPB) time, (**D**) DHCA time. NS: not statistically significant, ** *p* ≤ 0.01.

Table 2 shows the surgical and PCICU outcomes for included neonates and infants and compares outcomes between those with normal and those with abnormal microarrays. STS score was lower in the abnormal microarray group, with median values of 1.4 (IQR 0.5, 2.4) for the normal microarray and 0.8 (IQR 0.4, 1.5) for the abnormal microarray (*p* < 0.0001) [Figure 2A]. The abnormal microarray group had a larger proportion of patients in STAT categories 1 through 3. In comparison, the normal microarray group had a higher proportion of patients in STAT categories 4 and 5 [Figure 2B]. The cardiopulmonary bypass (CPB) time was shorter in the abnormal microarray group, with median values of 104 min (IQR 83, 132.25) for the normal microarray and 87 min (IQR 70.75, 118.25) for the abnormal microarray (*p* = 0.005) [Figure 2C]. The distribution of DHCA time was less variable in the abnormal microarray group, with a median of 0 min (IQR 0, 0), compared with a median of 0 min (IQR 0, 20.25) in the normal microarray group (*p* < 0.005) [Figure 2D]. The results of CPB time and DHCA time were consistent with the abnormal microarray group undergoing less complicated cardiac surgery with lower STAT categories. Days on total parental nutrition (TPN) had a lower trend in the abnormal microarray group, with median values for normal microarray of 10 days (IQR 3, 19) and abnormal microarray of five days (IQR 0, 16.5) (*p* = 0.019). Hospital LOS showed a median of 26 days (IQR 15, 43) for the normal microarray group and 25 days (IQR 6.75, 42.25) for the abnormal microarray group (*p* = 0.18). Both CICU LOS and mechanical ventilation days had a trend of shorter LOS in numerical changes without statistical significance in the abnormal microarray group, with medians of nine days (IQR 3, 19.3) and 2.7 days (IQR 0, 9.35), respectively. The usage of sub-ambient mixture had a trend of being less frequently required in the abnormal microarray group, with 6% requiring it vs. 12% in the normal microarray group (*p* = 0.083). Other measured outcomes include mortality at discharge and mortality at the time of 31 December 2023, the incidence of stroke during index hospitalization and to date of the study, the incidence of extracorporeal membrane oxygenation (ECMO) support during the index hospitalization, post-operative bleeding, unexpected re-operative intervention, incidence of hemorrhage in the first year of life, incidence of seizures during index hospitalization and to the date of the study, incidence of chylothorax, incidence of pulmonary hypertension, incidence of tracheostomy, incidence of necrotizing enterocolitis (NEC), incidence of deep vein thrombosis (DVT) during the index hospitalization, the necessity of tube feeds at discharge, and G-tube dependence at either one year or up to the year 2023 had no detectable difference between groups.

We investigated the differences between the normal and abnormal microarray groups for each investigated outcome after adjusting for the STAT category. Regression analysis demonstrated the abnormal microarray group had a higher chance of needing gastrostomy tube (G-tube) support during their lifetime [Table 3].

We further performed the first subgroup analysis and identified the cohort of patients who required an index operation as neonates or within the first 30 days of life. This included 158 total neonates. The cohort was 58% female, and 74% of the included neonates had normal microarray results. No neonates in this cohort were born before 33 weeks of gestational age, and 93% of neonates in both the normal and abnormal microarray groups were born after 37 weeks of gestational age. Supplemental Table S3 shows the demographic details of this cohort. Extracardiac anomalies, fundamental diagnoses, and primary procedures remained distinct between the normal and abnormal microarray groups and required their index operation within the first 30 days of life. Despite the difference in fundamental diagnosis and procedure performed between groups, there were no detectable differences in all measured outcomes [Supplemental Table S4].

The second subgroup analysis compared outcomes between normal and abnormal microarray groups in the sub-population of patients who presented with a diagnosis of VSD, AVSD, aortic stenosis/hypoplasia, or TOF. One hundred and forty-two neonates and infants were included in this subgroup analysis. Supplemental Table S5 shows the demographics and characteristics of this cohort. Forty-six percent of included infants were female, and 80% were born at a gestational age between 37 and 41 weeks. Twenty-eight percent of patients with normal microarrays in this subgroup analysis had extracardiac anomalies, compared with 33% in the abnormal microarray group (*p* = 0.63). Patients in the normal microarray group had an incidence of VSD, TOF, AVSD, and aortic stenosis/hypoplasia of 15%, 29%, 14%, and 40%, respectively. The incidence of VSD, TOF, AVSD, and aortic stenosis/hypoplasia in the abnormal microarray group was 29%, 20%, 38%, and 15%, respectively. DHCA time (*p* < 0.005) was once again significantly lower in the abnormal microarray subgroup [Supplemental Table S6]. Median DHCA time for the abnormal microarray group was 0 (IQR 0, 0), and median days on TPN for the abnormal microarray group was 0 (IQR 0, 8). On the other hand, only 15% of neonates and infants with normal microarrays in the subgroup required G-tube placement in their lifetime, whereas 33% of the abnormal microarray group have had G-tubes placed to date (*p* = 0.024). Other outcomes, including mortality, hospital LOS, CICU LOS, days on mechanical ventilation, and ECMO cannulation, showed no statistical difference between the two groups, indicating that patients with abnormal microarrays had comparable non-interior outcomes in relatively simple CHDs with straightforward procedures.

## 5. Discussion

Our data suggest that microarray results are not sufficient to prognosticate negative clinical outcomes in the post-operative period for neonates or infants undergoing cardiac surgery in the first year of life. The trend of the incidence of extracardiac anomalies and the difference in fundamental diagnoses and primary procedures across groups were expected, given the known associations of well-described genetic syndromes—such as Trisomy 21—with these factors. As with the prior study mentioned above, which examined associations between abnormal microarrays and clinical outcomes, this study again demonstrated no significant difference in mortality at discharge between the two groups [17]. Additionally, this study showed no difference between the two groups in long-term survival (more than four years). This is notably different from what has been described in previous studies that examined only pathogenic CNVs but may be attributable to the wider array of mutations and the more frequent smaller CNV sizes observed in the abnormal microarray results of this study [Supplemental Table S1]. Long-term neurocognitive outcomes were not investigated in this study. Still, there was no significant difference between the two groups in medical neurologic outcomes, such as the incidence of seizures and stroke to date. Notably, the results of this study suggest that abnormal microarray findings in infants are associated with lower STS scores, bypass times, and DHCA times. This trend is also observed in CICU LOS, mechanical ventilation days, and sub-ambient therapy, but these differences are not statistically significant. One hypothesis to explain this phenomenon is that children with more complex congenital heart defects may have more complex genetic etiologies that can be captured by microarray analysis, or that patients with abnormal microarrays had demise in utero [22]. This is reflected in the incidence of single-ventricle pathology between the normal and abnormal microarray groups in this study [Table 1]. Many institutions, including ours, have begun to adopt additional testing strategies or alternative first-line screening tools—such as whole-exome sequencing (WES)—to elucidate this idea. One limitation of this study is that the study period predated our current use of more advanced screening tools such as WES. Given the significant differences in primary diagnoses between the two groups in this study, a subgroup analysis was performed to assess only the four diagnoses most common in the abnormal microarray group. This subgroup analysis again showed significantly lower DHCA time for the abnormal microarray group, with essentially no differences in any other post-operative outcome. It must be acknowledged, though, that this study was subject to some degree of selection bias due to the lack of protocolized testing for all new CHD patients at our institution during the study period, which led to the exclusion of several otherwise healthy patients who were postnatally diagnosed with a VSD. While this single outcome-focused study does not suggest a change in clinical practice based on microarray results, it argues that further investigation into the prognostic power of genetic screening tools is warranted. Subsequently, this study also suggests that genetic screening results should not be used to exclude children from future trials investigating CHD interventions and outcomes. On the contrary, future outcomes-focused investigations should utilize newer genetic screening tools, such as whole-exome or whole-genome sequencing, to further our ability to optimize care for young patients with congenital heart disease.

## 6. Conclusions

In this single-center retrospective study, microarray results are insufficient to support clinically impactful prognostication for immediate postoperative care or to identify markers in the first few years of life. It does suggest that children with copy number variants (CNVs) should not be excluded from future trials investigating CHD interventions and outcomes. Our data support the need for additional testing, such as whole-exome or whole-genome sequencing, to guide clinical intervention.

## Figures and Tables

**Table 1:** Demographics and characteristics.

	Total	Normal Microarray	Abnormal Microarray	*p*-Value
	304	196 (0.64)	108 (0.36)	
**FEMALE**	125 (0.41)	79 (0.40)	46 (0.42)	0.885
**GESTATION AGE**				0.053
<33 WEEKS	8 (0.03)	7 (0.04)	1 (0.01)	
34–36 WEEKS	44 (0.14)	24 (0.12)	20 (0.18)	
37–41 WEEKS	252 (0.83)	165 (0.84)	87 (0.81)	
**BIRTH WEIGHT**				0.054
<2 KG	22 (0.07)	13 (0.07)	9 (0.08)	
2–3 KG	121 (0.40)	74 (0.38)	47 (0.43)	
3–4 KG	148 (0.49)	97 (0.50)	51 (0.47)	
>4 KG	12 (0.04)	11 (0.06)	1 (0.01)	
**RACE**				0.39
CAUCASIAN	252 (0.83)	163 (0.83)	89 (0.82)	
BLACK	34 (0.11)	22 (0.11)	12 (0.11)	
OTHER	4 (0.01)	1 (0.01)	3 (0.03)	
NOT DISCLOSED	14 (0.05)	10 (0.05)	4 (0.04)	
**EXTRACARDIAC ANOMALIES**	82 (0.27)	43 (0.22)	39 (0.36)	0.03
**FUNDAMENTAL DIAGNOSES**				<0.005
VSD	31 (0.10)	11 (0.06)	20 (0.18)	
TRUNCUS	13 (0.04)	7 (0.04)	6 (0.05)	
TGA	22 (0.07)	21 (0.10)	1 (0.01)	
TOF	35 (0.12)	21 (0.11)	14 (0.13)	
AVSD	36 (0.12)	10 (0.05)	26 (0.24)	
ASD	3 (0.01)	1 (<0.01)	2 (0.02)	
AORTIC STENOSIS/HYPOPLASIA	40 (0.13)	30 (0.15)	10 (0.09)	
EBSTEIN’S ANOMALY	5 (0.02)	4 (0.02)	1 (0.01)	
TAPVR	15 (0.05)	13 (0.07)	2 (0.02)	
DOUBLE OUTLET RIGHT VENTRICLE	16 (0.05)	14 (0.07)	2 (0.02)	
SINGLE VENTRICLE, RIGHT (HLHS)	42 (0.14)	34 (0.17)	8 (0.07)	
SINGLE VENTRICLE, LEFT (HRHS)	17 (0.06)	15 (0.08)	2 (0.02)	
INTERRUPTED OF AORTIC ARCH	6 (0.02)	2 (0.01)	4 (0.04)	
PA/PS	20 (0.06)	12 (0.06)	8 (0.07)	
OTHER	3 (0.01)	1 (<0.01)	2 (0.02)	
**PRIMARY PROCEDURE**				<0.005
ASD +/− VSD REPAIR	39 (0.13)	16 (0.08)	23 (0.21)	
AVSD REPAIR	34 (0.11)	7 (0.04)	27 (0.25)	
AORTIC ARCH REPAIR	49 (0.16)	33 (0.17)	16 (0.15)	
TRUNCUS REPAIR	13 (0.04)	7 (0.04)	6 (0.06)	
ARTERIAL SWITCH	32 (0.10)	30 (0.15)	2 (0.02)	
MODIFIED BT SHUNT (+/− NORWOOD)	47 (0.15)	39 (0.20)	8 (0.07)	
CONE PROCEDURE	3 (0.01)	2 (0.01)	1 (0.01)	
STARNES PROCEDURE	2 (<0.01)	2 (0.01)	0	
TOF REPAIR	32 (0.10)	22 (0.11)	10 (0.09)	
GLENN/SVC TO PA CONDUIT	11 (0.04)	9 (0.05)	2 (0.02)	
TAPVR REPAIR	13 (0.04)	11 (0.06)	2 (0.02)	
RV TO PA CONDUIT	10 (0.03)	4 (0.02)	6 (0.06)	
RVOT REPAIR	9 (0.03)	6 (0.03)	3 (0.03)	
CENTRAL SHUNT	3 (0.01)	3 (0.02)	0	
KAWASHIMA	2 (<0.01)	2 (0.01)	0	
OTHER	5 (0.02)	3 (0.02)	2 (0.02)	

**Table 2:** CICU and surgical outcomes.

	Total	Normal Microarray	Abnormal Microarray	*p*-Value
	N = 304	N = 196	N = 108	
**CICU LOS (DAYS) ^A^**	12 (3.75, 20)	13 (5, 20.25)	9 (3, 19.3)	0.064
**HOSPITAL LOS (DAYS) ^A^**	25 (10, 43)	26 (15, 43)	25 (6.75, 42.25)	0.180
**STS ^A^**	0.8 (0.5, 1.9)	1.4 (0.5, 2.4)	0.8 (0.4, 1.5)	**<0.005**
**STAT CATEGORY**				
**1**	52 (0.17)	23 (0.12)	29 (0.27)	**0.003**
**2**	68 (0.22)	45 (0.23)	23 (0.22)	0.78
**3**	53 (0.17)	27 (0.14)	26 (0.24)	0.045
**4**	88 (0.29)	65 (0.33)	23 (0.21)	0.063
**5**	43 (0.14)	36 (0.18)	7 (0.06)	**0.006**
**DHCA TIME (MINUTES) ^A^**	0 (0, 9)	0 (0, 20.25)	0 (0, 0)	**<0.005**
**BYPASS TIME (MIN) ^A^**	97.5 (74, 125)	104 (83, 132.25)	87 (70.75, 118.25)	**0.005**
**MECHANICAL VENTILATION (DAYS) ^A^**	4 (0.66, 9.5)	4.7 (1, 9.6)	2.7 (0, 9.35)	0.108
**TPN (DAYS) ^A^**	9 (0, 18)	10 (3, 19)	5 (0, 16.5)	0.019
**MORTALITY AT DISCHARGE ^B^**	9 (0.03)	4 (0.02)	5 (0.05)	0.202
**MORTALITY IN 2023 ^B^**	22 (0.07)	15 (0.08)	7 (0.06)	0.705
**ECMO ^B^**	52 (0.17)	36 (0.18)	16 (0.15)	0.431
**POST-OP BLEEDING ^B^**	16 (0.05)	11 (0.06)	5 (0.05)	0.713
**UNEXPECTED REOPERATIVE INTERVENTION ^B^**	18 (0.06)	14 (0.07)	4 (0.04)	0.224
**STROKE (DURING HOSPITALIZATION) ^B^**	22 (0.07)	16 (0.08)	6 (0.06)	0.401
**STROKE (LIFETIME INCIDENCE) ^B^**	28 (0.09)	20 (0.1)	8 (0.07)	0.419
**HEMORRHAGE IN 1ST YEAR ^B^**	23 (0.08)	17 (0.09)	6 (0.06)	0.325
**SEIZURE (DURING HOSPITALIZATION) ^B^**	7 (0.02)	4 (0.02)	3 (0.03)	0.681
**SEIZURE (LIFETIME INCIDENCE) ^B^**	27 (0.09)	19 (0.1)	8 (0.07)	0.494
**CHYLOTHORAX ^B^**	47 (0.015)	30 (0.15)	17 (0.16)	0.920
**SUBAMBIENT MIXTURE ^B^**	32 (0.11)	25 (0.12)	7 (0.06)	0.083
**PULMONARY HYPERTENSION ^B^**	14 (0.05)	8 (0.04)	6 (0.06)	0.557
**TRACH (DURING HOSPITALIZATION) ^B^**	17 (0.06)	12 (0.06)	5 (0.05)	0.587
**NECRPTOZON ENTEROCOLITIS ^B^**	21 (0.07)	15 (0.08)	6 (0.06)	0.490
**DVT (DURING HOSPITALIZATION) ^B^**	60 (0.20)	38 (0.19)	22 (0.20)	0.873
**TUBE FEEDS AT DISCHARGE ^B^**	175 (0.58)	119 (0.60)	56 (0.52)	0.209
**G-TUBE AT 1 YR ^B^**	97 (0.32)	58 (0.30)	39 (0.36)	0.243
**G-TUBE (LIFETIME INCIDENCE) ^B^**	101 (0.33)	59 (0.30)	42 (0.39)	0.119

A—Median (IQR); *p*-value obtained via Wilcoxon rank-sum test. B—Incidence (%); *p*-value obtained via chi-squared test. DVT: deep vein thrombosis, ECMO: Extracorporeal membrane oxygenation, G-tube: Gastrostomy tube.

**Table 3:** Regression analysis for CICU and surgical outcomes adjusted by stat categories.

VARIABLES	ODDS RATIO	95% CI	*T*-TEST STAT	*p*-VALUE
**GESTATIONAL AGE (WEEKS)**	−0.11	(−0.569, 0.349)	−0.468	0.64
**BIRTH WEIGHT (KG)**	−0.14	(−0.297, 0.018)	−1.741	0.083
**CICU LOS (DAYS)**	5.476	(−0.078, 11.029)	1.932	0.054
**HOSPITAL LOS (INDEX)**	10.426	(0.143, 20.709)	1.987	0.048
**STS**	−0.041	(−0.202, 0.119)	−0.506	0.613
**DHCA TIME (MINS)**	−2.168	(−5.784, 1.448)	−1.175	0.241
**BYPASS TIME (MINS)**	−9.545	(−25.79, 6.701)	−1.152	0.25
**CROSS-CLAMP TIME (MINS)**	3.545	(−4.327, 11.417)	0.883	0.378
**VENTILATOR DURATION (HOURS)**	43.078	(−41.699, 127.855)	0.996	0.32
**MECHNICAL VENTILATION (DAYS)**	2.208	(−2.92, 7.337)	0.844	0.399
**TPN (DAYS)**	3.988	(−0.949, 8.926)	1.583	0.114
**VARIABLES**	**ODDS RATIO**	**95% CI**	**Z-TEST STAT**	***p*** **-VALUE**
**SEX**	0.939	(0.568, 1.553)	−0.245	0.807
**EXTRACARDIAC ANOMALIES**	1.756	(1.021, 3.021)	2.034	0.042
**MORTALITY (DISCHARGE)**	3.723	(0.916, 15.122)	1.838	0.066
**LIVE/DEATH AT 2023**	0.886	(0.331, 2.374)	−0.24	0.81
**ECMO**	1.677	(0.786, 3.577)	1.338	0.181
**BLEEDING REQUIRED RE-OPERATION**	1.229	(0.387, 3.895)	0.35	0.727
**UNEXPECTED INTERVENTION**	0.675	(0.205, 2.223)	−0.647	0.518
**STROKE (INDEX)**	1.015	(0.365, 2.822)	0.028	0.978
**STROKE (LIFETIME)**	1.061	(0.43, 2.623)	0.129	0.897
**CHYLOTHORAX**	1.016	(0.517, 2)	0.047	0.963
**SUB-AMBIENT MIXTURE**	1.075	(0.397, 2.915)	0.143	0.886
**PULMONARY HYPERTENSION**	1.534	(0.493, 4.775)	0.738	0.46
**TRACH ON DISCHARGE (INDEX HOSPITALIZATION)**	1.218	(0.399, 3.715)	0.346	0.729
**NECROTIZING ENTEROCOLITIS**	0.915	(0.329, 2.541)	−0.171	0.864
**DEEP VEIN THROMBOSIS (INDEX)**	1.471	(0.783, 2.765)	1.2	0.23
**G-TUBE (FIRST YEAR)**	2.045	(1.173, 3.567)	2.523	0.012
**G-TUBE (LIFETIME)**	2.204	(1.271, 3.822)	2.813	**0.005**
**TUBE FEEDS ON DISCHARGE**	1.063	(0.624, 1.808)	0.224	0.823

## Data Availability

Data are contained within the article and Supplementary Material.

## Supplementary Materials

The following supporting information can be downloaded at: https://www.mdpi.com/article/10.3390/genes17080910/s1.
