# Supplementary material for: Genetic Variation and Association with Post-Operative Outcomes for Neonates and Infants in the Cardiac Intensive Care Unit
Source: Genes (Basel). 2026 Jul 31;17(8):910. doi: 10.3390/genes17080910 (PMC13511873; doi:10.3390/genes17080910)
Supplement: Supplementary file 1 [file genes-17-00910-s001.zip › genes-4357726-supplementary.pdf]

SUPPLEMENTAL TABLE S1. GENETIC DATA FROM SCREENING MICROARRAY TESTING

GENETIC FINDING	# Children affected	Areas Affected
**45X**	1	-
**HOMOZYGOSITY (ROH)**	5	-
**TRANSLOCATION**	2	-
**TRISOMY 21**	48	-
**XXY**	2	-
**DUPLICATION**	10	15q13.3<br>13q31.1<br>1q21.1<br>22q11.2<br>2q13
**DELETION**	19	10q22<br>11q14<br>11q22<br>16p12<br>17p12<br>19q13<br>1p36<br>1q21<br>2p16<br>2q13<br>3p26<br>3q29<br>5p13<br>6q16<br>7q11<br>7q31<br>8p23
**22Q11.2 DELETION**	15	-
**MULTIPLE COPY NUMBER VARIANTS**	5	15q11<br>17p13<br>Xp22<br>22q11.2<br>16p13<br>1p22<br>8q11<br>15q13
**UNKNOWN**	1	Testing obtained at birth facility and reported 'abnormal' in documentation

SUPPLEMENTAL TABLE S2. WHOLE EXOME SEQUENCING (WES) RESULTS	
**MICROARRAY FINDINGS**	WES RESULTS
**NORMAL**	negative
**NORMAL**	negative
**NORMAL**	negative
**NORMAL**	negative
**NORMAL**	multiple point mutations
**NORMAL**	point mutation
**NORMAL**	point mutation
**NORMAL**	point mutation
**NORMAL**	point mutation
**NORMAL**	point mutation
**NORMAL**	point mutation
**NORMAL**	point mutation
**NORMAL**	point mutation
**NORMAL**	point mutation
**NORMAL**	point mutation
**NORMAL**	mitochondrial point mutation
**XXY**	negative
**TRISOMY 21**	negative
**TRISOMY 21**	homozygosity in HFE and PRF1
**TRISOMY 21**	point mutation
**DUPLICATION 15Q13.3**	point mutation
**DELETION 2Q13**	point mutation
**HOMOZYGOSITY**	multiple point mutations
**TRANSLOCATION**	translocation & multiple point mutations

SUPPLEMENTAL TABLE S3. DEMOGRAPHIC DATA FOR PATIENTS WITH INDEX OPERATIONS IN NEONATAL PERIOD (LESS THAN OR EQUAL TO 30-DAY-OLD)				
	Total	Normal Microarray	Abnormal Microarray	p-value
	158	117 (0.74)	41 (0.26)	
**FEMALE**	58 (0.37)	44 (0.38)	14 (0.34)	0.86
**GESTATION AGE**				0.64
<33 WEEKS	0	0	0	
34-36 WEEKS	11 (0.07)	8 (0.07)	3 (0.07)	
37 – 41 WEEKS	147 (0.93)	109 (0.93)	38 (0.93)	
**BIRTH WEIGHT**				0.15
<2 KG	2 (0.01)	2 (0.02)	0	
2-3 KG	50 (0.32)	35 (0.30)	15 (0.37)	
3-4 KG	97 (0.61)	71 (0.61)	26 (0.63)	
>4 KG	9 (0.06)	9 (0.08)	0	
**EXTRACARDIAC ANOMALIES**	28 (0.18)	15 (0.13)	13 (0.32)	0.012
**FUNDAMENTAL DIAGNOSES**				<0.01
VSD	0	0	0	
TRUNCUS ARTERIOSUS	11 (0.07)	5 (0.04)	6 (0.15)	
TGA	18 (0.11)	17 (0.14)	1 (0.02)	
TOF	8 (0.05)	5 (0.04)	3 (0.07)	
AVSD	2 (0.01)	1 (0.01)	1 (0.02)	
ASD	0	0	0	
AORTIC STENOSIS/ HYPOPLASIA	31 (0.20)	24 (0.21)	7 (0.17)	
EBSTEIN'S ANOMALY	3 (0.02)	2 (0.02)	1 (0.02)	
TAPVR	11 (0.07)	10 (0.08)	2 (0.05)	
DORV	10 (0.06)	9 (0.08)	1 (0.02)	
SINGLE VENTRICLE, RIGHT	30 (0.19)	28 (0.24)	2 (0.05)	
SINGLE VENTRICLE, LEFT	9 (0.06)	8 (0.07)	1 (0.02)	
INTERRUPTED OF AORTIC ARCH	6 (0.04)	2 (0.02)	4 (0.10)	
PA/PS	11 (0.07)	6 (0.05)	5 (0.12)	
OTHER	1 (0.01)	0	1 (0.02)	
**PRIMARY PROCEDURE**				<0.01
ASD +/- VSD REPAIR	4 (0.03)	3 (0.03)	1 (0.02)	
AVSD REPAIR	0	0	0	
AORTIC ARCH REPAIR	38 (0.24)	26 (0.22)	12 (0.29)	
TRUNCUS REPAIR	11 (0.07)	5 (0.04)	6 (0.14)	
ARTERIAL SWITCH	27 (0.17)	25 (0.21)	2 (0.05)	
MODIFIED BT SHUNT (+/- NORWOOD)	43 (0.27)	35 (0.30)	8 (0.20)	
CONE PROCEDURE	1 (0.01)	0	1 (0.02)	
STARNES PROCEDURE	2 (0.01)	2 (0.02)	0	
TOF REPAIR	6 (0.04)	5 (0.04)	1 (0.02)	
GLENN/SVC TO PA CONDUIT	2 (0.01)	1 (0.01)	1 (0.02)	
TAPVR REPAIR	10 (0.06)	8 (0.07)	2 (0.05)	
RV TO PA CONDUIT	6 (0.04)	1 (0.01)	5 (0.12)	
RVOT REPAIR	1 (0.01)	1 (0.01)	0	
CENTRAL SHUNT	3 (0.02)	3 (0.02)	0	
KAWASHIMA	0	0	0	
OTHER	4 (0.03)	2 (0.02)	2 (0.05)	

SUPPLEMENTAL TABLE S4. CICU AND SURGICAL OUTCOMES FOR PATIENTS WITH INDEX OPERATIONS IN NEONATAL PERIOD				
	Total	Normal Microarray	Abnormal Microarray	p-value
	N = 158	N = 117	N = 41	
**CICU LOS (DAYS)[A]**	16 (11, 24)	15 (11, 23)	17 (13, 25)	0.39
**HOSPITAL LOS (DAYS)[A]**	31 (22, 46)	31 (21, 43)	31 (25, 54)	0.26
**STS[A]**	1.6 (1.05, 2.4)	1.6 (1.17, 2.7)	1.6 (0.72, 2.4)	0.28
**DHCA TIME (MINUTES)[A]**	0 (0,26.8)	0 (0, 28)	0 (0, 23)	0.30
**BYPASS TIME (MIN)[A]**	108 (84, 141)	109 (84, 142)	102 (76, 124)	0.32
**MECHANICAL VENTILATION (DAYS)[A]**	7.1 (3.7, 12.7)	6.6 (3.7, 11.5)	8.7 (5, 15.5)	0.12
**TPN (DAYS)[A]**	13 (9, 22)	13 (9, 21)	16 (10, 25)	0.21
**MORTALITY AT DISCHARGE[B]**	7 (0.04)	4 (0.03)	3 (0.07)	0.54
**MORTALITY IN 2023[B]**	18 (0.11)	14 (0.12)	4 (0.10)	0.92
**ECMO[B]**	41 (0.265)	31 (0.26)	10 (0.24)	0.95
**POST-OP BLEEDING[B]**	13 (0.08)	11 (0.09)	2 (0.05)	0.56
**UNEXPECTED REOPERATIVE INTERVENTION[B]**	12 (0.07)	11 (0.09)	1 (0.02)	0.27
**STROKE (DURING HOSPITALIZATION)[B]**	18 (0.11)	15 (0.13)	3 (0.07)	0.50
**STROKE (LIFETIME INCIDENCE)[B]**	22 (0.14)	17 (0.14)	5 (0.12)	0.91
**HEMORRHAGE IN 1ST YEAR[B]**	19 (0.12)	14 (0.12)	5 (0.12)	1
**SEIZURE (DURING HOSPITALIZATION)[B]**	5 (0.03)	4 (0.03)	1 (0.02)	1
**SEIZURE (LIFETIME INCIDENCE)[B]**	16 (0.10)	12 (0.10)	4 (0.10)	1
**CHYLOTHORAX[B]**	28 (0.18)	22 (0.19)	6 (0.14)	0.71
**HYPOXIC MIXTURE[B]**	27 (0.17)	20 (0.17)	7 (0.17)	1
**PULMONARY HYPERTENSION[B]**	4 (0.03)	2 (0.02)	2 (0.05)	0.59
**TRACH (DURING HOSPITALIZATION)[B]**	11 (0.07)	8 (0.07)	3 (0.07)	1
**NEC[B]**	11 (0.07)	9 (0.08)	2 (0.05)	0.80
**DVT (DURING HOSPITALIZATION)[B]**	37 (0.23)	25 (0.21)	12 (0.29)	0.41
**TUBE FEEDS AT DISCHARGE[B]**	115 (0.73)	86 (0.74)	29 (0.70)	0.89
**G-TUBE AT 1 YR[B]**	58 (0.37)	39 (0.33)	19 (0.46)	0.19
**GTUBE (LIFETIME INCIDENCE)[B]**	60 (0.38)	40 (0.34)	20 (0.49)	0.14

A- MEDIAN (IQR); P-VALUE OBTAINED VIA WILCOXON-RANK SUM TEST.

B- INCIDENCE (%); P-VALUE OBTAINED VIA CHI-SQUARED TEST.

SUPPLEMENTAL TABLE S5. DEMOGRAPHIC DATA FOR CHILDREN WITH VSD, AVSD, AS, AND TOF				
	Total	Normal Microarray	Abnormal Microarray	p-value
	142	72 (0.51)	70 (0.49)	
**FEMALE**	66 (0.46)	33 (0.46)	33 (0.47)	1
**GESTATION AGE**				0.002
<33 WEEKS	4 (0.03)	3 (0.04)	1 (0.01)	
34-36 WEEKS	25 (0.18)	9 (0.12)	16 (0.24)	
37 – 41 WEEKS	113 (0.80)	60 (0.83)	53 (0.75)	
**BIRTH WEIGHT**				0.022
<2 KG	12 (0.08)	6 (0.08)	6 (0.09)	
2-3 KG	59 (0.42)	24 (0.34)	35 (0.5)	
3-4 KG	64 (0.45)	35 (0.48)	29 (0.41)	
>4 KG	6 (0.04)	6 (0.08)	0 (0)	
**EXTRACARDIAC ANOMALIES**	43 (0.30)	20 (0.28)	23 (0.33)	0.63
**FUNDAMENTAL DIAGNOSES**				
VSD	31 (0.22)	11 (0.15)	20 (0.29)	
TOF	35 (0.25)	21 (0.29)	14 (0.20)	
AVSD	36 (0.25)	10 (0.14)	26 (0.38)	
AORTIC STENOSIS / HYPOPLASIA	40 (0.28)	30 (0.40)	10 (0.15)	
**PRIMARY PROCEDURE**				
ASD +/- VSD REPAIR	39 (0.13)	11 (0.15)	20 (0.29)	
AVSD REPAIR	34 (0.11)	7 (0.10)	27 (0.38)	
AORTIC ARCH REPAIR	49 (0.16)	31 (0.42)	12 (0.18)	
MODIFIED BT SHUNT (+/- NORWOOD)	47 (0.15)	2 (0.03)	0 (0)	
TOF REPAIR	32 (0.10)	18 (0.25)	9 (0.13)	
GLENN/SVC TO PA CONDUIT	11 (0.04)	0 (0)	1 (0.01)	
RV TO PA CONDUIT	10 (0.03)	2 (0.03)	1 (0.01)	
RVOT REPAIR	9 (0.03)	1 (0.01)	0 (0)	

SUPPLEMENTAL TABLE S6. CICU AND SURGICAL OUTCOMES FOR CHILDREN WITH VSD, AVSD, AS, AND TOF				
	Total	Normal Microarray	Abnormal Microarray	p-value
	N = 142	N = 72	N = 70	
**CICU LOS (DAYS)[A]**	4 (3, 13)	6.5 (3, 13)	3 (2, 12.25)	0.19
**HOSPITAL LOS (DAYS) [A]**	15.5 (6, 30)	18 (7, 33)	9 (5, 28)	0.23
**STS [A]**	0.8 (0.3, 1.4)	0.8 (0.35, 1.4)	0.8 (0.2, 0.8)	0.04
**DHCA TIME (MINUTES) [A]**	0 (0, 12.25)	0 (0, 28)	0 (0, 0)	**<0.005**
**BYPASS TIME (MIN) [A]**	87.5 (68.2, 109)	93 (64, 109)	84 (70, 111.8)	0.56
**MECHANICAL VENTILATION (DAYS) [A]**	1 (0, 4.7)	1.8 (0, 5)	0.4 (0, 4.7)	0.14
**TPN (DAYS) [A]**	1 (0, 10)	5 (0, 12)	0 (0, 8)	0.03
**MORTALITY AT DISCHARGE[B]**	1 (0.01)	1 (0.01)	0 (0)	1
**MORTALITY IN 2023[B]**	4 (0.03)	3 (0.03)	1 (0.01)	0.63
**ECMO[B]**	7 (0.05)	5 (0.07)	2 (0.03)	0.46
**POST-OP BLEEDING[B]**	2 (0.01)	0 (0)	2 (0.03)	0.46
**UNEXPECTED REOPERATIVE INTERVENTION[B]**	3 (0.02)	2 (0.03)	1 (0.01)	1
**STROKE (DURING HOSPITALIZATION) [B]**	3 (0.02)	2 (0.03)	1 (0.01)	1
**HEMORRHAGE IN 1ST YEAR[B]**	4 (0.03)	4 (0.05)	0 (0)	0.14
**SEIZURE (DURING HOSPITALIZATION) [B]**	1 (0.01)	0 (0)	1 (0.01)	0.99
**CHYLOTHORAX[B]**	18 (0.13)	7 (0.09)	11 (0.16)	0.41
**SUBAMBIENT MIXTURE[B]**	1 (0.01)	1 (0.01)	0 (0)	1
**PULMONARY HYPERTENSION[B]**	6 (0.04)	3 (0.04)	3 (0.04)	1
**TRACH (DURING HOSPITALIZATION) [B]**	2 (0.01)	1 (0.14)	1 (0.01)	1
**NEC[B]**	7 (0.05)	2 (0.03)	5 (0.07)	0.42
**DVT (DURING HOSPITALIZATION) [B]**	17 (0.12)	8 (0.11)	9 (0.13)	0.95
**TUBE FEEDS AT DISCHARGE[B]**	60 (0.42)	33 (0.46)	27 (0.38)	0.48
**GTUBE (LIFETIME INCIDENCE) [B]**	34 (0.24)	11 (0.15)	23 (0.33)	0.024

A- MEDIAN (IQR); P-VALUE OBTAINED VIA WILCOXON-RANK SUM TEST.
B- INCIDENCE (%); P-VALUE OBTAINED VIA CHI-SQUARED TEST.
